# Development and Validation of a Prognostic Nomogram for Predicting Cancer-Specific Survival in Patients With Lymph Node Positive Bladder Cancer: A Study Based on SEER Database

**DOI:** 10.3389/fonc.2022.789028

**Published:** 2022-02-03

**Authors:** Xiangpeng Zhan, Ming Jiang, Wen Deng, Xiaoqiang Liu, Luyao Chen, Bin Fu

**Affiliations:** ^1^ Department of Urology, The First Affiliated Hospital of Nanchang University, Nanchang, China; ^2^ Jiangxi Institute of Urology, Nanchang, China

**Keywords:** lymph node-positive, bladder cancer, SEER, prognosis, nomogram

## Abstract

**Purpose:**

To construct a prognostic model to predict the cancer-specific survival (CSS) for bladder cancer patients with lymph node-positive.

**Patients and Methods:**

We enrolled 2,050 patients diagnosed with lymph node-positive bladder cancer from the Surveillance Epidemiology and End Results (SEER) database (2004–2015). All patients were randomly split into development cohort (n = 1,438) and validation cohort (n = 612) at a ratio of 7:3. The univariate and multivariate Cox regression analysis were performed to identify prognostic factors. A nomogram predicting CSS was established based on the results of multivariate Cox analysis. Its performance was evaluated by calibration curves, the receiver operating characteristic (ROC) curves, and the concordance index (C-index). Internal verification was performed in the validation cohort. The Kaplan–Meier method with the log-rank test was applied in the different risk groups.

**Results:**

The nomogram incorporated summary stage, tumor size, chemotherapy, regional nodes examined and positive lymph nodes. The C-index of the nomogram in the development cohort was 0.716 (0.707–0.725), while the value of the C-index was 0.691 (0.689–0.693) in the validation cohort. The AUC of the nomogram was 0.803 for 3-year and 0.854 for 5-year in the development cohort, while was 0.773 for 3-year and 0.809 for 5-year in the validation cohort. Calibration plots for 3-year and 5-year CSS showed good concordance. Significant differences were observed between high, medium, and low risk groups (*P <*0.001).

**Conclusions:**

We have established a prognostic nomogram providing an accurate individualized probability of cancer-specific survival in bladder cancer patients with lymph node-positive. The nomogram could contribute to patient counseling, follow-up scheduling, and selection of treatment.

## Introduction

Bladder cancer (BC) is a common malignancy globally, with an estimated 500,000 new cases and 200,000 deaths worldwide in 2018 (1, 2). In addition, bladder cancer is also a severe and heterogeneous disease with a poor prognosis, especially for those patients with lymph node-positive (3). A retrospective study showed that approximately 25–30% of BC patients undergoing radical cystectomy presented with lymph node-positive after pathologic examination. Moreover, only a 25% disease-free survival rate was observed in these patients (4). Several retrospective studies had confirmed the poor prognosis of the higher recurrence and poorer survival rate in node-positive patients compared with those without (4–7). For example, a survey demonstrated that up to 70–80% of node-positive patients experienced disease recurrence, while this data was only 30% in patients with negative pathological nodes.

Over the course of the past years, some urologists were committed to stratifying patients with lymph node metastasis because a few studies suggested that a part of node-positive patients was still potentially curable (8). Jensen revealed better prognosis was observed in patients with a single node-positive compared with those patients with multiple (9). Meanwhile, a more prolonged overall survival (OS) and cancer-specific survival (CSS) were seen in patients staged N1 in comparison to patients with more extensive node involvement, according to the results of some retrospective studies (10). All these studies intended to meticulously stratify node-positive patients and pick out patients with better prognosis to take more suitable treatment. The eighth edition TNM system of the American Joint Committee on Cancer (AJCC), which divided node-positive patients into N1, N2, and N3 stages was used widely to simply evaluate the prognosis (2). However, lack of high accuracy and vital tumor characteristics like the number of positive nodes were its limitations. When compared with the conventional TNM system, a few studies suggested that the number of positive nodes seemed to be a more promising predictor of the outcome for node-positive BC patients (3, 11). Thus, it is imperative to build an exact model to evaluate the prognosis of BC patients with node-positive.

Nomogram is a visible and trustworthy statistical prediction tool, which was utilized widely to provide tailored individual prognostic information. Nomogram was composed of fundamental variables like demographics, tumor characteristics, and treatment features (12). Rink had constructed a nomogram that included gender, T stage, margin status, LN-density, and adjuvant chemotherapy to predict recurrence and cancer-specific survival for patients with a single lymph node metastasis (13). Meanwhile, a nomogram integrating multiple molecular markers was constructed to access disease recurrence and cancer-specific mortality for BC patients with locally advanced and node-positive (14). However, these models failed to obtain high accuracy (C-index 0.63 and 0.66 for Rink’s model) and incorporate variables not easily available. Moreover, the models were not specially designed for all bladder cancer patients with positive lymph nodes. To our knowledge, it is the first study to construct a prognostic nomogram to predict cancer-specific survival (CSS) in all node-positive patients.

In our study, we searched patients with node-positive and collected all information available from the Surveillance, Epidemiology, and End Results (SEER) database from 2004 to 2015. We were committed to establishing a prognostic nomogram that incorporated significant factors to estimate the CSS and make direct decisions on treatment for those patients with node-positive. In addition, the performance of the nomogram was evaluated, and an assessment of applicability with internal verification was also performed in this study.

## Patients and Method

### Data Source and Patient Selection

All patients were collected from the Surveillance, Epidemiology, and End Results (SEER), which included particular patient demographic and cancer information of the US population. The following inclusion criteria were applied: (1) diagnosed from 2004 to 2014; (2) number of positive lymph nodes more than 1; (3) surgical approach to confirm positive lymph nodes: partial cystectomy, radical cystectomy and pelvic exenteration (Code 30,50,61,62,63,64,71,72,73,74,80). (4) Histology behavior: Transitional cell carcinoma. The exclusion criteria were as follows: (1) race unknown (n = 8); (2) grade unknown (n = 241); (3) Tx (n = 15); (4) Nx (n = 4); (5) chemotherapy unknown (n = 814); (6) tumor size unknown (n = 598); (7) marital status unknown (n = 78); (8) M1 and Mx stage (n = 317).

### Variables Defined and End Point

The variables in the selected cohorts included: demographic characteristics (age, sex, race, marital status), tumor characteristics (tumor size, grade, histology, T stage, N stage, summary stage), treatment information (chemotherapy and radiotherapy), and other variables (regional nodes examined and positive lymph nodes). The prime endpoint in this study was cancer-specific mortality (CSM), which referred to the death of bladder cancer.

For conveniently analyzing, we had processed some variables in the SEER database. Some continuous variables, namely, age, tumor size, regional nodes examined and positive lymph nodes were transformed into categorical variables: age (<60, 60–70, 70–80, >80); tumor size (<3 cm, ≥3 cm); positive lymph nodes (1, 2–10, >10). Sex was divided into male and female, and race included white, black, others which contained American, Indian, Alaska, Native, Asian, and Pacific Island. We defined marital status as married, separated, divorced or widowed (SDW), and single. Our study only was committed to the common histology with transitional cell carcinoma (TCC) and papillary transitional cell carcinoma (PTCC). Grades I and II were combined, considering the small sample size. According to the sixth edition of the AJCC stages, precise information on the TMN system was recorded in this study.

### Statistical Analysis

We randomly split the study population into development and validation cohorts based on the ratio of 7:3. The Student’s t-test and Chi-square test were performed for continuous and categorical variables, respectively, to explore the baseline characteristics of patients in the two groups. Categorical variables were presented as frequencies and their proportions, while continuous variables were the mean ± Standard Deviation (SD). In the development cohort, the univariate Cox regression analysis was applied to recognize potential significant prognostic factors. They were incorporated in the multiple Cox proportional hazards regression model when their *P*-value was under 0.05. All results were shown as hazards ratios (HR) and 95% confidence intervals (95%CI).

A nomogram incorporated the selected variables from the multiple Cox model, and the critical *P*-value was 0.05. the nomogram was built for visualized prediction of 3- and 5-year survival probability in the development cohort. We used Harrell’s concordance-index (C-index) and the receiver operating characteristic (ROC) curves with the calculated area under the curve (AUC) to assess the performances of the model. Moreover, the consistency of predicted and actual outcomes of 3- and 5-year survival time was evaluated by the calibration plots, and it was performed with the package of *rms* in Rstudio. Patients in the development cohort were divided into three levels of risk group based on the total obtaining points. Meanwhile, the Kaplan–Meier method with the log-rank test was applied to analyze the differences of CSS between the three risk groups. SPSS 22.0 (IBM Corp, Armonk, NY) and R version 3.6.3 (https://cran.r-project.org/bin/windows/base/old/3.6.3) were utilized for all statistic analysis.

## Results

### Characteristics of Study Population

Finally, 2,050 patients with lymph node-positive were enrolled in our study, and 1,438 patients (70%) were distributed into the development cohort while 612 patients (30%) into the validation cohort. Baseline demographical and clinicopathological characteristics of the study population are shown in **Table 1**. There were statistical differences between development and validation cohorts on the grade (*P* = 0.013), and patients in the development cohort tended to have a higher proportion of distant stage (43.9% vs 30.6%, *P <*0.001). Statistical differences on other variables between the two groups were failed to observe. The 3- and 5-year CSS rates were 43.17% (n = 885) and 37.56% (n = 770) in total cohort, respectively, while 43.6% (n = 627) and 37.83% (n = 544) in the development cohort, respectively. The mean survival time was 34.16, 35.12, and 31.9 months in the total cohort, development cohort, and validation cohort, respectively.

**Table 1:** Baseline demographical and clinicopathological characteristics of patients.

Characteristics	Total cohort *N* (%)	Development cohort *N* (%)	Validation cohort *N* (%)	*p-*value
**Number of patients**	2,050	1,438 (70%)	612 (30%)	
**Median age** (25th–75th percentile)	67.5 (62–77.5)	67.5 (62–77.5)	67.5 (62–77.5)	0.328
**Mean age**	67.85	67.68	68.25	0.253
**Age**				0.310
<60	463 (22.6%)	332 (23.1%)	131 (21.4%)	
60–70	683 (33.3%)	472 (32.8%)	211 (34.5%)	
70–80	595 (29.0%)	428 (29.8%)	167 (27.3%)	
>80	309 (15.1%)	206 (14.3%)	103 (16.8%)	
**Sex**				0.092
Female	544 (26.5%)	397 (27.6%)	147 (24.0%)	
male	1,506 (73.5%)	1,041 (72.4%)	465 (76.0%)	
**Race**				0.935
White	1,806 (88.1%)	1,265 (88.0%)	541 (88.4%)	
Black	137 (6.7%)	98 (6.8%)	39 (6.4%)	
others	107 (5.2%)	75 (5.2%)	32 (5.2%)	
**Marital status**				0.854
Married	1,275 (62.2%)	900 (62.6%)	375 (61.3%)	
SDW	524 (25.6%)	364 (25.3%)	160 (26.1%)	
Single	251 (12.2%)	174 (12.1%)	77 (12.6%)	
**Histology**				0.793
TCC	1,452 (70.8%)	1,021 (71.0%)	431 (70.4%)	
PTCC	598 (29.2%)	417 (29.0%)	181 (29.6%)	
**Grade**				0.013^※^
Grade I or II	31 (1.5%)	27 (1.9%)	4 (0.7%)	
Grade III	576 (28.1%)	422 (29.3%)	154 (25.2%)	
Grade IV	1,443 (70.4%)	989 (68.8%)	454 (74.2%)	
**T stage**				0.809
T1	25 (1.2%)	16 (1.1%)	9 (1.5%)	
T2	394 (19.2%)	281 (19.5%)	113 (18.5%)	
T3	1,046 (51.0%)	736 (51.2%)	310 (50.7%)	
T4	585 (28.5%)	405 (28.2)	180 (29.4%)	
**N stage**				0.834
N1	978 (47.7%)	680 (47.3%)	298 (48.7%)	
N2	1,033 (50.4%)	730 (50.8%)	303 (49.5%)	
N3	39 (1.9%)	28 (1.9%)	11 (1.8%)	
**Summary Stage**				<0.001^※^
Regional	1,232 (60.1%)	807 (56.1%)	425 (69.4%)	
Distant	818 (39.9%)	631 (43.9%)	187 (30.6%)	
**Tumor size**				0.216
<3 cm	670 (32.7%)	482 (33.5%)	188 (30.7%)	
>3 cm	1,380 (67.3%)	956 (66.5%)	424 (69.3%)	
**Chemotherapy**				0.411
No	823 (40.1%)	569 (39.6%)	254 (41.5%)	
Yes	1,227 (59.9%)	869 (60.4%)	358 (58.5%)	
**Radiotherapy**				0.391
No	1,930 (94.1%)	1,358 (94.4%)	572 (93.5%)	
Yes	120 (5.9%)	80 (5.6%)	40 (6.5%)	
**Regional nodes examined**				0.455
<10	603 (29.4%)	420 (29.2%)	183 (29.9%)	
10–20	784 (38.2%)	544 (37.8%)	240 (39.2%)	
20–30	348 (17.0%)	241 (16.8%)	107 (17.5%)	
>30	315 (15.4%)	233 (16.2%)	82 (13.4%)	
**Positive lymph nodes**				0.721
1	850 (41.5%)	588 (40.9%)	262 (42.8%)	
2–10	1,086 (53.0%)	769 (53.5%)	317 (51.8%)	
>10	114 (5.6%)	81 (5.6%)	33 (5.4%)	
**Survival time(month)**				
mean	34.16	35.12	31.9	0.056
median (25th–75th percentile)	20 (10–47)	20 (10–49)	18 (9–42)	0.051

Other race, American/Indian/Alaska/Native/Asian/Pacific Islands; SDW, separated, divorced or widowed; TCC, Transitional cell carcinoma; PTCC, papillary Transitional cell carcinoma.

※:Statistical significance.

### Prognostic Factors of Node-Positive Patients in Development Cohort

Ultimately, five factors, namely, summary stage, tumor size, chemotherapy, regional nodes examined and positive lymph nodes were selected from the multivariate cox model. Five independent factors were determined as following: distant stage (HR = 3.927, 95%CI: 3.393–4.545, *P <*0.001); tumor size >3 cm (HR = 1.240, 95%CI: 1.075–1.430, *P* = 0.003); receiving chemotherapy (HR = 0.684, 95%CI: 0.594–0.787, *P <*0.001); regional nodes examined of 20–30 (HR = 0.784, 95%CI: 0.638–0.963, *P* = 0.021), >30 (HR = 0.673, 95%CI: 0.545–0.830, *P <*0.001); positive lymph nodes of 2–10 (HR = 1.234, 95%CI: 1.018–1.496, *P* = 0.032), >10 (HR = 1.687, 95%CI: 1.219–2.336, *P* = 0.002) (**Table 2**).

**Table 2:** Univariate and multivariate regression analyses for CSM.

Characteristics	Univariate analysis	*p-*value	Multivariate analysis	*p-*value
	HR (95%CI)		HR (95%CI)	
**Age**				
<60	Ref.		Ref.	
60–70	1.082 (0.917–1.277)	0.351	0.884 (0.737–1.060)	0.184
70–80	1.390 (1.178–1.640)	<0.001^※^	1.062 (0.884–1.277)	0.519
>80	1.815 (1.493–2.205)	<0.001^※^	1.140 (0.908–1.433)	0.259
**Sex**				
Female	Ref.			
male	1.112 (0.973–1.270)	0.119		
**Race**				
White	Ref.			
Black	1.113 (0.861–1.439)	0.412		
others	0.898 (0.671–1.201)	0.468		
**Marital status**				
Married	Ref.			
SDW	1.133 (0.973–1.319)	0.108		
Single	1.108 (0.908–1.350)	0.312		
**Histology**				
TCC	Ref.			
PTCC	0.868 (0.752–1.003)	0.055		
**Grade**				
Grade I or II	Ref.			
Grade III	0.862 (0.548–1.357)	0.522		
Grade IV	0.782 (0.501–1.220)	0.278		
**T stage**				
T1	Ref.		Ref.	
T2	1.306 (0.576–2.960)	0.523	0.879 (0.386–2.001)	0.759
T3	2.455 (1.097–5.494)	0.029^※^	1.377 (0.612–3.097)	0.440
T4	3.800 (1.693–8.531)	0.001^※^	1.718 (0.760–3.882)	0.193
**N stage**				
N1	Ref.		Ref.	
N2	1.397 (1.225–1.593)	<0.001^※^	1.056 (0.877–1.272)	0.563
N3	1.599 (1.020–2.506)	0.041^※^	1.066 (0.663–1.713)	0.792
**Summary Stage**				
Regional	Ref.		Ref.	
Distant	4.413 (3.827–5.091)	<0.001^※^	3.927 (3.393–4.545)	<0.001^※^
**Tumor size**				
<3 cm	Ref.		Ref.	
>3 cm	1.339 (1.165–1.539)	<0.001^※^	1.240 (1.075–1.430)	0.003^※^
**Chemotherapy**				
No	Ref.		Ref.	
Yes	0.705 (0.619–0.804)	<0.001^※^	0.684 (0.594–0.787)	<0.001^※^
**Radiotherapy**				
No	Ref.		Ref.	
Yes	1.378 (1.058–1.794)	0.017^※^	1.285 (0.981–1.684)	0.069
**Regional nodes examined**				
<10	Ref.			
10–20	0.918 (0.785–1.072)	0.279	0.916 (0.782–1.073)	0.279
20–30	0.782 (0.641–0.955)	0.016	0.784 (0.638–0.963)	0.021^※^
>30	0.684 (0.557–0.839)	<0.001^※^	0.673 (0.545–0.830)	<0.001^※^
**Positive lymph nodes**				
1				
2–10	1.456 (1.269–1.671)	<0.001^※^	1.234 (1.018–1.496)	0.032^※^
>10	2.014 (1.535–2.643)	<0.001^※^	1.687 (1.219–2.336)	0.002^※^

CSM, Cancer-specific mortality; Other race, American/Indian/Alaska/Native/Asian/Pacific Islands; SDW, separated, divorced or widowed; TCC, Transitional cell carcinoma, PTCC, papillary Transitional cell carcinoma.

※:Statistical significance.

### Prognostic Nomogram for OS

**Figure 1:**
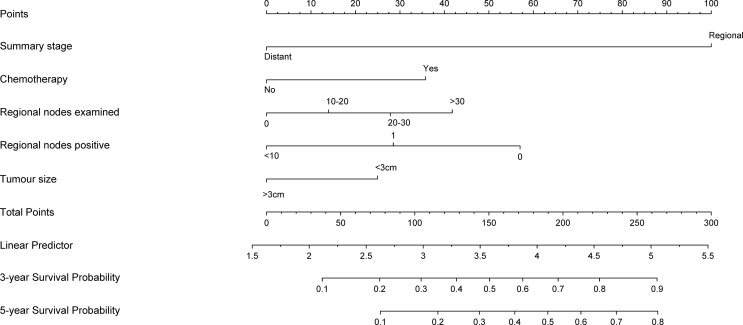
Nomogram predicting 3- and 5-year cancer-specific survival probability for bladder cancer patients with lymph node-positive. use: locate patient values at each axis. Draw a vertical line to the “Point” axis to determine how many points are attributed for each variable value. Sum the points for all variables. Locate the sum on the “Total Points” line. Draw a vertical line towards the 3Yrs.Surv. Prob. and 5Yrs.Surv. Prob, Prob. axes to determine respectively the 3-, and 5-year survival probabilities.

A nomogram predicted the 3- and 5-year CSS of node-positive patients based on the Cox regression models (**Figure 1**). All variables in the nomogram were assigned a corresponding score of 0 to 100 based on the contribution to this nomogram (**Table 3**). Each patient could obtain a total score by adding scores in every subgroup. The nomogram revealed that the summary stage was the most significant contributor to the prognosis model of CSS.

**Table 3:** Nomogram scoring system.

Variables	Points	Variables	Points
**Summary stage**		Regional nodes positive	
Regional	100	1	94.04
Distant	0	2–10	47.02
**Chemotherapy**		3	0
No	0	Regional nodes examined	
Yes	65.52	<10	0
**Tumor size**		10–20	18.67
<3 cm	25	20–30	37.35
≥3 cm	0	>30	56.02
			
3-Year CSM probability	Points	5-Year CSM probability	Points
0.1	39	0.1	78
0.2	73	0.2	119
0.3	107	0.3	148
0.4	129	0.4	169
0.5	151	0.5	190
0.6	173	0.6	218
0.7	198	0.7	238
0.8	224	0.8	264
0.9	262	0.9	

SDW, separated, divorced or widowed; STBS, Systemic therapy before surgery; STAS, Systemic therapy after surgery; IST, Intraoperative systemic therapy; CSM, Cancer-specific mortality.

### Validation of the Nomogram

**Figure 2:**
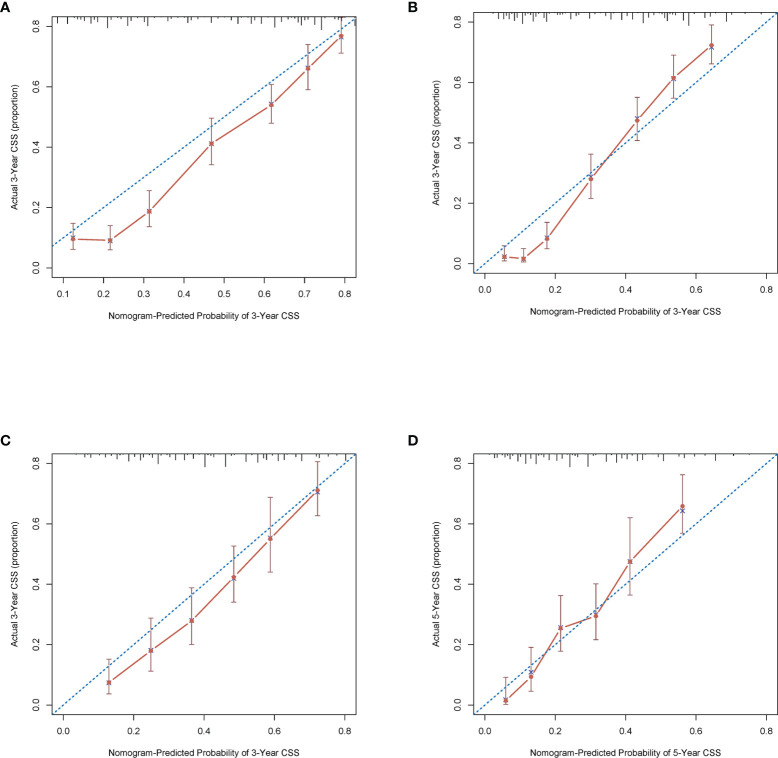
Calibration plots of the nomogram describing 3- **(A)** and 5-year **(B)** CSS in the development cohort; 3- **(C)** and 5-year **(D)** CSS in the validation cohort.

**Figure 3:**
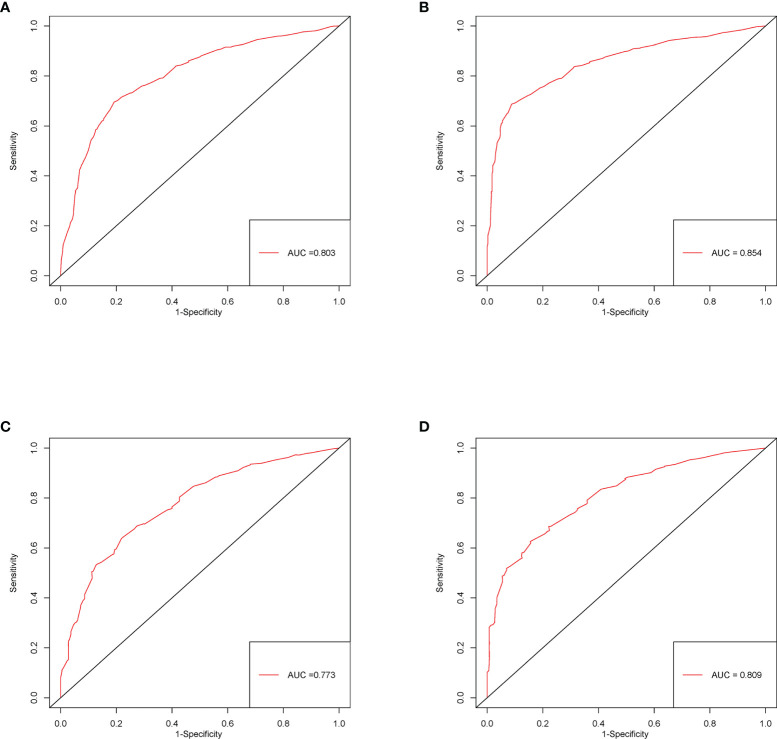
ROC curves of the nomogram predicting 3-year **(A)** and 5-year **(B)** CSS in the development cohort; 3- **(C)** and 5-year **(D)** CSS in the validation cohort.

The C-index of this nomogram for CSS was 0.716 (0.707–0.725) in the development cohort, which was more significant than 0.605 of the TNM system (*P <*0.05). Meanwhile, the discriminative ability of the nomogram was evaluated by ROC curves. The AUC of the nomogram was significantly higher than the TMN system both for 3-year (0.803 vs 0.675) and 5-year (0.854 vs 0.669) CSS prediction (all *P <*0.05) (**Figures 3A, B** **)**. The calibration plots of the development cohort for 3-year and 5-year all demonstrated good agreement between actual observations and predicted outcomes (**Figures 2A, B** **)** All these results suggested that better performance of our model in comparison to the traditional TNM system. In addition, internal verification of the nomogram was performed in the validation cohort to evaluate the applicability. The C-index of this nomogram was 0.691 (0.689–0.693), and AUC was 0.773 and also 0.809 for 3-year and 5-year, respectively (**Figures 3C, D** **)**. The calibration curve of the validation cohort all gained good correlation between nomogram prediction and actual outcomes, especially for 5-year prediction. The results of internal validation suggested that this nomogram had satisfying applicability for node-positive patients (**Figures 2C, D** **)**.

### Survival Curve for Nomogram

**Figure 4:**
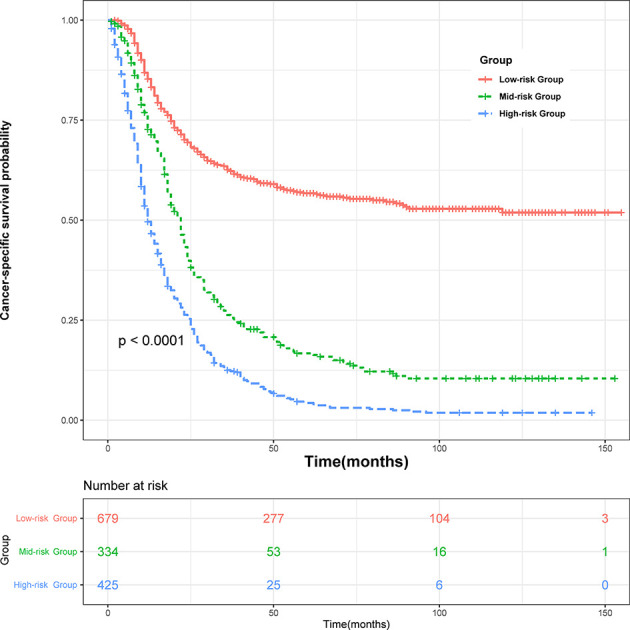
Survival curves stratified by the score calculated by the nomogram. low-risk group (score >198); medium group (score 148–198); high-risk group (score <148).

All variables in the nomogram have authorized a score based on the contribution to the CSS, and we provided a corresponding score of 3-year and 5-year cancer-specific mortality probability, respectively. The lymph node-positive patients were divided into three risk subgroups according to the total points obtained: Low risk group: >198; medium risk group: 148–198; high risk group: <148. As **Figure 4** showed, significant differences in CSS were observed between the three risk subgroups (*P <*0.001).

## Discussion

Lymph node-positive bladder cancer was considered as a severe stage associated with a high recurrence rate and mortality rate (8, 14). However, a part of patients with node metastasis still could be curable after active treatment (13). In addition, with the development of the treatment for bladder cancer patients, a lot of novel treatments such extend lymph node dissection, neoadjuvant chemotherapy, and targeted molecular therapy were proposed, and they acquired better prognosis possible for part node-positive patients (8, 14, 15). However, the prognostic stratification for patients with node-positive is still lacking. Therefore, it is urgent to establish an accurate and suitable predictive model for patients with lymph node metastasis.

This study comprehensively explored the effect of all factors available in the SEER database in CSS in node-positive patients. Meanwhile, we constructed and internally validated a relatively accurate and discriminating nomogram for the prediction of CSS by incorporating variables from the multivariate cox model. This approach produced a relatively easy and accurate tool, which only incorporated the significant variables associated with survival outcome but without sacrificing accuracy. The final survival nomogram yielded highly accurate prediction far exceeded the accuracy of individual predictors. In addition, the other advantage of nomogram over standard multivariate regression model was providing the individual probability of survival outcome at specific time points instead of a relative risk concept. Meanwhile, using Harrell’s concordance index, which was a global measure of model accuracy to evaluate the accuracy of the nomogram, was also the advantage compared to conventional Cox regression models (12, 16–18). Furthermore, different levels of risk groups could be constructed based on the points of the nomogram, and individual patient counseling and follow-up scheduling were tailored for different risk groups (16).

We had compared our nomogram with the traditional AJCC TNM classification on clinical performance by the C-index and AUC. The results showed that our model obtained a greater C-index and AUC composed to the TNM system in the development cohort. Bruins et al. retrospectively enrolled 146 node-positive patients to evaluate the effect of the TNM system and failed to obtain differences on overall survival and disease-free survival (DFS) between patients staging N1–3 (19). Meanwhile, Jensen had constructed a nomogram based on 381 pN1 patients, namely, gender, T stage, margin status, LN density, and adjuvant chemotherapy. However, only focusing on pN1 patients and excluding patients with neoadjuvant chemotherapy limited its applicability in all node-positive patients. Moreover, the C-index of the model was 0.66 and 0.63, respectively, and it seemed to not be enough to satisfy the accuracy of the model (9). A nomogram in the combination of multiple molecular markers incorporating p53, pRB, p21, and p27 applied for predicting recurrence and cancer-specific survival (CSS) in pT3–4 or node-positive patients (14). Nevertheless, adding the molecular markers to the model failed to significantly improve the performance of outcome prediction (3.9% for recurrence, 4.3% for CSS) (20). Moreover, the application of molecular marker was still limited on account of ambiguously effect and expensive cost. The nomogram in this study had a great clinical performance in CSS prediction and variables incorporated relatively easily accessible in most hospitals. In detail, the good discriminative ability and accuracy of the nomogram were confirmed with the relatively high C-index and AUC of 3-year and 5-year in development and validation cohorts. The calibration curves also revealed a perfect consistency between the prediction of the nomogram and the actual outcome.

This novel nomogram for CSS probability prediction incorporated five factors, which included summary stage, regional nodes positive, tumor size, regional nodes examined, and chemotherapy. Studies suggested that a number of positive nodes seem to be a more promising predictor of outcome in node-positive patients than the conventional TNM system (3). In addition, some researchers found significant differences in disease outcome between patients with one and more nodes positive (9, 13, 21). Meanwhile, a retrospective study with 244 node-positive patients obtained the result that the 10-year disease-free survival rate in patients with eight or fewer positive nodes was significantly higher than those with greater than eight positive nodes. The degree of the number of positive nodes had been confirmed to strongly associate with prognosis in node-positive patients. Furthermore, receiving chemotherapy was shown as a protective factor for patients with node-positive. Several retrospective studies enrolling bladder cancer patients with node-positive observed higher overall survival and cancer-specific survival rates in patients with chemotherapy than those patients without (9, 15, 21). Therefore, chemotherapy might be a suitable and meaningful treatment for patients with node-positive.

Several significant advantages were worth noting in this study. First, it is the first study, up to our knowledge, to perform a prognostic nomogram for the prediction of CSS for all bladder cancer patients with lymph node-positive. Then, the number of patients in this study was relatively great and enough to construct a prognostic nomogram with good performance (n = 2,050). Finally, the variables in the nomogram were easily available in most hospitals, and the good applicability was obtained in our nomogram. Meanwhile, we divided study population into three risk groups based on the prognostic nomogram, and it was easier to detect patients with worse survival outcomes. Nevertheless, some limitations in this study should be noticed. First of all, this is a retrospective study based on the SEER database, which means the results of this study were inevitably influenced by selection biases. In addition, we excluded patients with unknown variable information, and it was also a significant source of selection biases. Second, there were some limitations in the SEER database. Such as the SEER database collected massive information of patients from multiple regions and hospitals, and it seemed impossible to balance the differences in treatment and pathological evaluation standards. Moreover, some vital factors like drugs of chemotherapy and course of treatment of radiotherapy, which were also vital for node-positive patients, were lacking in the SEER database. Simultaneously, novel treatment such as target therapy is a growing field, and they need more research to verify the effect (8). Finally, although internal verification was performed in the validation cohort, the result of this verification method was not perfect because the patients in the development and validation came from the same database. Therefore, a large prospective clinical trial was demanded for external validation.

## Conclusion

The study based on the SEER database revealed several demographics, lymph node characteristics, and therapeutic features, which were significantly associated with the cancer-specific survival of bladder cancer patients with lymph node-positive. A prognostic nomogram was constructed and validated to predict the individualized probability of cancer-specific survival at the time of 3- and 5-year. The nomogram could contribute to patient counseling, follow-up scheduling, and selection of treatment. Nonetheless, external and prospective validation was demanded for widely applying.

## Data Availability Statement

The original contributions presented in the study are included in the article/supplementary material. Further inquiries can be directed to the corresponding authors.

## Ethics Statement

The data from SEER is publicly available and de-identified. This study was approved by the institutional. This study was approved by the institutional of the First Affiliated Hospital of Nanchang University.

## Author Contributions

All authors listed have made a substantial, direct, and intellectual contribution to the work and approved it for publication.

## Funding

This work was supported by grants from the National Natural Science Foundation of China (81960512).

## Conflict of Interest

The authors declare that the research was conducted in the absence of any commercial or financial relationships that could be construed as a potential conflict of interest.

## Publisher’s Note

All claims expressed in this article are solely those of the authors and do not necessarily represent those of their affiliated organizations, or those of the publisher, the editors and the reviewers. Any product that may be evaluated in this article, or claim that may be made by its manufacturer, is not guaranteed or endorsed by the publisher.
